# Pathology

**Published:** 1859-03

**Authors:** 


### PATHOLOGY.

### IV.—*On Apoplexy of the Cerebellum.* By DR. J. B. HILLAIRET.

FROM a considerable number of carefully reported cases, the material for which was offered to Dr. Hillairet in the *Hospice des Incurables*, at Paris, the author draws the following general conclusions in regard to the semeiology of apoplexy of the cerebellum:—"Between the different parts which compose the substance of the brain there exist such intimate relations, and, to a certain point, such a functional harmony, that it necessarily affects also the symptomatology of their diseases to a very marked degree. It is from this reason one of the most difficult tasks of diagnosis to distinguish the affections of the different parts of the brain. Until the present time, observations have not been sufficiently numerous to establish reliable characteristics for the differential diagnosis of apoplexy of the cerebrum and cerebellum. The following facts may, however, be considered as an endeavor to commence the solution of this question."

From the cases reported by the author, it results that, although apoplexy of the cerebrum and cerebellum have many symptoms in common, as, for instance, cephalalgia, vertigo, stupefaction, tinnitus aurium, hemiplegia, etc., apoplexy of the cerebellum is still generally marked by a collection of peculiar symptoms:— 1. Apoplexy of the cerebellum may occur under two forms: one with a slow, though steadily progressing course, from the time of the attack to the fatal termination; the other appearing suddenly, and with an extremely rapid course. The latter may even produce instantaneous death, which is not the case in apoplexy of the cerebrum. 2. In the first form the attack is not accompanied with loss of consciousness; in the second, if the fit is violent, consciousness may be lost in rare cases for a short time; but it always returns very soon. 3. In both forms the attack is accompanied by vomiting, which is repeated during the course of the disease, and lasts until death; sometimes it is incessant and unyielding; in the first form, however, it is more frequent than in the second. Vomiting does not appear to be a symptom of apoplexy of the cerebrum, as many authors state; it is, on the contrary, peculiar to apoplexy of the cerebellum as well as to any other affection which may produce an increase of the volume of this organ. The examination of many facts leads the author to the supposition that the symptom in question is owing to a slight compression, or to irritation of the par vagum, caused by the dilatation of the cerebellar substance which follows the hemorrrhage; from a similar cause vomiting is a frequent symptom in abscesses, tubercles, cysts, etc. of the cerebellum, while in softening, where the increase of the circumference of the organ is almost imperceptible, it is rarely met with. According to his own experience, and to facts furnished by extensive statistical researches, the author believes himself justified in stating that, contrary to the general opinion, spontaneous vomiting is a very rare symptom in those cases of apoplexy of the cerebrum which are not accompanied by any complication on the part of the cerebellum. While vomiting is only observed in one case out of thirty of apoplexy of the cerebrum, the proportion in apoplexy of the cerebellum is one to two and a half. 4. Somnolence and coma are common in the severer form, but the intellect is not destroyed on that account except a few hours before death; even in patients attacked by fulminant apoplexy of the cerebellum, consciousness often remains, for they are able to communicate their suffering by words and gestures at the moment of attack; they generally die very soon afterwards. In fulminant apoplexy of the cerebrum, on the contrary, the patients fall down senseless, without uttering a sound or making a sign, and have stertorous breathing. 5. The relaxation of the extremities is likewise a usual symptom of apoplexy of the cerebellum. The patients cannot maintain their equilibrium on standing up, but are capable of moving the lower extremities while lying down; they can likewise keep their limbs up in any position given to them. Hemiplegia is not as frequent as many authors state, and is met with only in a third of the cases. The author did not observe it in any of his cases. The paralysis always affects the side of the body opposite to the one in which the effusion has taken place. 6. The tendency to retrogressive or rotatory motion was observed by the author in only one instance. 7 Facial paralysis of the opposite side, as observed in apoplexy of the cerebrum, is only exceptionally met with in apoplexy of the cerebellum; among twenty-six cases the author found only one in which one corner of the mouth was distorted. Deviation of the tongue is also exceedingly rare; the speech is nevertheless commonly inarticulated and slow. The features bear a rather peculiar expression of stupidity and astonishment, accompanied by a staring gaze,—an expression worthy of notice in regard to diagnosis. This peculiarity usually disappears some time after the attack. 8. Sensibility remains intact in the severer apoplexy of the cerebellum; once the author observed transient hyperæsthesia in the course of the disease. Only at the approach of the agony of death the sensibility becomes more blunt and feeble, and seems to disappear with the increase of coma. The contrary takes place in apoplexy of the cerebrum, in which sensibility is generally lost from the moment of the attack. 9. As is the case with general sensibility, the senses also remain intact; the hearing, smell, and taste are changed only in exceptional cases; sometimes sight becomes feebler, or is even lost, if the processus cerebelli ad testes are injured or destroyed. Contraction and immobility of the pupil is the rule, dilatation the exception. 10. Convulsions do not occur in apoplexy of the cerebellum, except if the injury of the cerebellum is complicated with injuries of other parts of the central nervous system, or if the hemorrhage is followed by inflammation of the surrounding cerebellar tissue. 11. With exception of the vomiting mentioned above, the digestive organs suffer only slight or no disturbance; the bowels are mostly constipated; sometimes, however, the discharge is involuntary. The evacuation of urine is in cases of the first form voluntary at the commencement, but becomes involuntary toward the end of the malady; in the course of the second form it is involuntary. 12. The average duration of the disease is one and a half days. The slowness of respiration which soon becomes stertorous, is apparently owing as much to the relaxation of the whole muscular system, particularly of the intercostal muscles, as to the pressure to which the par vagum is subjected. It is probably due to these circumstances that the rapidly fatal termination of the disease is mostly dependent. Although death is the general consequence of apoplexy of the cerebellum, the possibility of a cure is not excluded.

As regards the causes of apoplexy of the cerebellum, they are essentially the same as those of apoplexy of the cerebrum. Like the latter, so also the former is most frequent between the sixty-first and seventy-seventh years of life, and is more common in men than in women. Of thirty cases of apoplexy of the cerebellum, eighteen occurred in males, and twelve in females.

The pathological changes are the same as those observed in the cerebrum. Hemorrhage into the centre of the cerebellar substance was most frequently met with; only in five cases was it confined to the surface. In five cases the worm only was attacked, and destroyed; in three the fourth ventricle was the seat of the hemorrhage, and in three other cases both hemispheres of the cerebellum were implicated at the same time. The clot of blood varied in size from that of a nut to that of a pigeon's egg; in some cases it was very considerable, and in one the affected hemisphere was converted into a pocket containing blood. In five cases the hemorrhage was preceded by softening; in some other cases cerebellitis was developed around the seat of the effusion; it was particularly in those cases in which complete convulsions or isolated spasms had been observed. In six cases extravasations were met with simultaneously in the cerebrum and cerebellum.

The treatment of the disease under consideration is of course the same as that of apoplexy of the cerebrum.—(*Archives Générales de Médecine*, 1858.)

### V.—*Observations on Pneumonia.* By DR. METZGER.

From forty-eight cases of pneumonia which he had observed in the clinic of Professor Pfeufer, in Munich, Dr. Metzger draws the following conclusions:—

1. There are certain signs observable in the course of pneumonia which constantly and on a certain day introduce a favorable crisis of the disease; *herpes labialis* is one of these signs. 2. A constant symptom also is the diminution of the chlorides in the urine, observed during the continuance of the fever and exudation, and the increase of the same during the process of resorption. 3. The secretion of urea, which is insignificant during the first days of the disease, reaches its maximum on the sixth day. 4. The diminution of temperature is preliminary to the decrease in the frequency of the pulse. 5. The intermittent character of the pulse indicates that the pulse is about to become slower, particularly if the temperature is at the same time diminishing; the presence of other bad symptoms may, however, render an unfavorable interpretation of this sign necessary. 6. During convalescence of pneumonia it is constantly observed, that the pulse becomes slower even underneath its normal standard. 7. Some of the observed cases show the possibility that double pneumonia may originate from morbid changes in the central organs of the nervous system.—(*Zeitschrift für Rationelle Medizin*, 1858, iv. 3.)

VI.—*On Rheumatism of the Diaphragm.* By Dr. Chenevier.

After reporting several cases of this disease, the author gives the following description of it:—The disease commences with a sudden pain at the points of attachment of the diaphragm, which produces a feeling of constriction at the base of the thorax, but is not augmented on pressure. Deep inspirations are impossible, and respiration is carried on only by the superior ribs. Percussion is normal, and auscultation does not reveal any change in the respiratory murmur, which is only somewhat weaker at the base of the thorax; there is no cough; sometimes, however, a painful hiccough. The abdominal organs offer no symptom of disease. The attack lasts from one to eight hours, and disappears then without leaving any trace. The prognosis is favorable. Rheumatism of the diaphragm is easily distinguished from inflammatory diseases of the lungs by the absence of the symptoms of the latter. It could only be mistaken for a neuralgic affection of neighboring organs, as, for instance, intercostal neuralgia; but it is sufficiently distinguished from it by the pain being felt particularly in the three characteristic points, while in the neuralgia just mentioned it is confined to one side. From angina pectoris it is distinguished by the peculiarity that the pain proceeds in this malady from the sternum and radiates on one side to the arm. In nervous asthma, which also commences with sudden difficulty of breathing, the peculiar feeling of constriction as well as the confinement of the respiratory movements to the superior ribs, is not noticed; the two latter symptoms are pathognomonic of rheumatism of the diaphragm.

The treatment of the disease consists in the application of cups, mustard poultices, anodyne embrocations, and chloroform; if it is obstinate, the endermatic application of morphia will be useful.—(*Gazette des Hôpitaux*, 1858, No. 35.)

VII.—*On the Non-Elimination of Odorous Substances by the Urine in Bright's Disease.* By M. de Beauvais. (Communicated to the *Académie des Sciences*, October 25th.)

Repeated clinical researches have proved to M. de Beauvais that odorous substances, fixed or volatile, do not pass by the urine in confirmed Bright's disease.

This circumstance is an exclusive, pathognomonic sign of the malady; it persists with the disease when the symptom of albuminuria is momentarily suppressed; it never exists in albuminuria arising from other causes, no matter how persistent this may be. It confirms the importance and the nature of the part taken by the cortical substance in the elaboration of the urine. It proves the distinct character of Bright's disease, and of the morbid transformations peculiar to it; and it reveals at the same time the serious and incurable character of the confirmed disease. In the absence of the albuminuria, the principal symptom, or of the characteristic dropsy, the absolute and incurable suppression of the passage of odors serves at the same time to indicate the diagnosis, the prognosis, and the treatment. In addition to this, the presence of the symptom is easily ascertained by administering the juice of asparagus, or the essence of turpentine.—(*Archives Générales*, December, 1858.)

VIII.—*A Case of Spontaneous Hydrophobia.* By DR. HENRICH.

F. K., thirty years of age, suffered on the twenty-ninth of May, 1857, of cephalalgia, which radiated from the forehead to the occiput, and of all the symptoms of a cold in the head. On the morning of the thirtieth he complained of chills, and distressing horripilations. Dr. Henrich examined the patient attentively, without finding in the throat or elsewhere a single sign of disease. In the evening he was called in great haste to the patient, whom he found sitting in the bed, the face bathed in perspiration, pale, and expressing terror; the eyes injected, brilliant, haggard; the voice hoarse, anxious, and broken. The patient complained of pain and constriction in the throat and chest, of intense thirst with impossibility to drink, and of dryness of the mouth. The respiratory movements were accelerated, superficial, and irregular; they became normal in the interval of the spasms, which followed each other rapidly; but when the throat became constricted, the patient seemed to suffocate, and carried the hand to the neck as if to remove an obstacle to respiration. The saliva flowed in great quantity from his mouth. Pulse ninety, and feeble. Pharynx a little reddened, and covered like the mouth with viscid mucus.

After earnest entreaties, Dr. Henrich finally succeeded in trying to overcome his violent horror of liquids; after having for a long time struggled against a convulsive contraction of the muscles of the forearm, he could finally bring a glass of water to his mouth, but hardly had the first few drops of the liquid touched his lips, when he was seized with a violent attack of suffocation. He threw his glass away with a gesture of despair, and taking refuge in the remotest part of the bed, cried out to take the water away; that he could not swallow; that he was suffocating.

In this condition he remained during the night. The impression of light or of a current of air exercised, however, no perceptible influence upon the spasms, and the vesicles of Morochetti were not found on the margin of the tongue. In spite of venesection, a blister on the chest, etc., all the symptoms were aggravated the next day; chloroform exasperated them; and they became less violent only for a few moments, after the patient had lost about a pound of blood through the wound made by the venesection, which had opened again; but soon they returned with greater intensity; tetanic convulsions and opisthotonos supervened, and the patient expired half an hour later. He had preserved the full power of his intellectual functions until tetanus came on.

On autopsy a very slight swelling of the base of the tongue was discovered; the pharynx was in a healthy condition; some pulmonary hypostasis, and two hemorrhagic suffusions in the mucous membrane of the stomach were found. All the other organs, the spinal marrow included, presented no alteration. The blood was black, liquid, and diffluent.

Dr. Henrich assured himself, by the most careful inquiries, that the patient had never been bitten by a dog, either mad or healthy, and that he did not believe himself at all attacked by hydrophobia. For three weeks previous, however, he was in low spirits, and without being otherwise sick, had a presentiment of his approaching death, as he said. He indulged, however, in excessive coïtus, (he was married and kept two mistresses,) and was troubled with grief and sorrow. To these two causes combined, the appearance of the terrible malady may be attributable.—(*Henke's Zeitschrift für Staatsarzneikunde,* 1858, p. 361.)

This case, which belongs to the third class of spontaneous hydrophobia of M. Chomel, (*Dict. de Méd.*, tome xv., 1837,) is, among all the published cases, one of the most characteristic. Cases of similar kind have been reported by MM. Ely, Burggreave, (*Gaz. des Hôpitaux,* 1854,) Lessmann, (*Preuss. Vereinszeitung,* 1854,) Bulley, (*Assoc. Med. Journ.*, 1854, Nov. 11,) and Putegnat, (*Journ. de Méd. de Bruxelles,* June, 1853.)—(*Gazette Hebdomad.*, 1858, 40.)

